# The Analgesic Effects of the Thoracic Paravertebral Block on Post-percutaneous Nephrolithotripsy: A Retrospective Study

**DOI:** 10.7759/cureus.60272

**Published:** 2024-05-14

**Authors:** Heitor JS Medeiros, Erica Gee, Aimee Pak, Vivian Hu, Lane Crawford, Sarah Razavi, T Anthony Anderson, A. Sassan Sabouri

**Affiliations:** 1 Anesthesia, Critical Care, and Pain Medicine, Massachusetts General Hospital, Boston, USA; 2 Anesthesiology, University of Oklahoma Health Sciences Center, Oklahoma City, USA; 3 Urology, Massachusetts General Hospital, Boston, USA

**Keywords:** opioid-related disorders, analgesia, nerve block, pain, percutaneous nephrolithotomy

## Abstract

Introduction: Percutaneous nephrolithotripsy (PCNL) is a minimally invasive procedure for treating large and complex kidney stones, often resulting in significant post-operative pain and increased opioid use. This study aims to compare pain scores between patients undergoing PCNL who did and did not receive a preoperative single-shot thoracic paravertebral block (PVB) at the post-anesthesia care unit (PACU) as the primary outcome. Secondary outcomes were patient-controlled analgesia (PCA) usage on post-operative day 1 (POD 1), total opioid consumption on PACU and POD 1, and post-operative nausea and vomiting (PONV).

Methods: A retrospective cohort study was conducted on the medical records of 341 patients who underwent PCNL from July 2014 to April 2016 in a single major academic center. PVB was administered at thoracic levels T7-9 using a volume of 20 cc of bupivacaine, ranging from 0.25% to 0.5%, to achieve the desired analgesic effect.

Results: After excluding 34 patients, the study included 123 in the no block (NB) group and 149 in the regional anesthesia (RA) group. There were no differences in demographics, including age, sex, weight and height, BMI, and indication for PCNL. The results revealed that the RA group experienced a statistically significant reduction in PCA usage in both crude and adjusted models (adjusted logistic regression analysis: OR = 0.19, 95% CI = 0.05-0.60; p = 0.008). However, there were no significant changes in total opioid consumption, pain scores, or incidents of PONV.

Conclusion: The retrospective analysis did not reveal any discernible advantage in pain management associated with the use of PVB for post-PCNL analgesia, except for reducing the percentage of PCA narcotics used. Future investigations with larger sample sizes and meticulous control for surgical indications and complexity are imperative to accurately assess the efficacy of this block in the context of post-PCNL surgery.

## Introduction

Nephrolithiasis affects about 12% of the world's population, with 600,000 cases annually in America​​. Emergency department visits for this condition increased by 20% from 2005 to 2009 [1-3]. Nephrolithiasis is associated with high morbidity and potential mortality [4] and a greater than 30% recurrence rate within a decade [5]. Surgical and interventional approaches such as percutaneous nephrolithotripsy (PCNL) ureteroscopy and shock-wave lithotripsy have been used to treat nephrolithiasis. PCNL is a minimally invasive inpatient procedure under general anesthesia to treat nephrolithiasis. PCNL is increasingly used for the treatment of large and complex stones and has better stone-free rates compared to ureteroscopy and shock-wave lithotripsy [6,7]. PCNL entails the introduction of wide-diameter, inflexible scopes through a cut on the side of the body into the kidney's central cavity to crush stones and extract resulting fragments [8]. This procedure requires creating a percutaneous tract, distension of the renal capsule and pelvicalyceal system, and placement of nephrostomy tubes. This often results in severe post-operative pain, which increases the need for opioid analgesics and further increases the risk of opioid-related adverse events, as well as delayed hospital discharge. Thus, interest in the use of regional anesthesia (RA) techniques to improve perioperative pain control for patients undergoing PCNL has grown [9,10]. The need for neuraxial analgesia, including thoracic epidural, has been decreased in PCNL patients as these patients are usually discharged in less than a day. There are limited reports of the utility of other RA techniques to control postoperative pain in these patients, including thoracic paravertebral block (PVB) [11]. The thoracic PVB is obtained by injecting the local anesthetic mixture into the paravertebral space. It provides dense and profound somatic and visceral pain control without interfering with patient ambulation or urination and, therefore, has been used more frequently for outpatient or next-day discharge procedures [12].

## Materials and methods

Methods

This is a retrospective cohort study approved by the Review Board (#2015P002436). Electronic and paper medical charts were reviewed from 308 adult patients undergoing PCNL from July 2014 to April 2016. The authors conducted data analysis at the same center where the procedures were performed. Inclusion criteria encompassed adults (≥18 years) of both genders undergoing PCNL who received a thoracic PVB for analgesia within the specified timeframe. Exclusion criteria comprised patients undergoing surgical procedures other than PCNL, those administered RA besides a PVB perioperatively, and individuals with incomplete or missing data.

Procedure for the RA group

Before induction and surgery, patients were positioned prone, and the standard American Society of Anesthesiology (ASA) monitoring was applied - non-invasive blood pressure, five-way cardioscopy, and pulse oximetry. The skin over the appropriate vertebral level (T7-9) was thoroughly disinfected. Using a 21G block needle (Pajunk Medical Systems, Alpharetta, GA) and aided by a Sonosite Edge Ultrasound Device (Sonosite, Bothell, WA) equipped with a linear array 5-10 MHz probe, the needle was inserted perpendicularly. The needle was guided until it reached the paravertebral space, confirmed by depression of pleural and the presence of local anesthetic in the paravertebral space in different levels, under ultrasound image. A volume of 20 cc of bupivacaine, ranging from 0.25% to 0.5%, was injected to achieve the desired analgesic effect.

No block (NB) group

Patients who did not receive a PVB were assigned to the NB group, receiving the standard intravenous analgesia regimen for pain management during and after PCNL.

Outcomes

The primary outcome is the pain scores in the Post-Anesthesia Care Unit (PACU) postoperatively, utilizing the visual analog scale (VAS), a validated tool [13] ranging from 0 to 10, where 0 represents no pain and 10 indicates the worst possible pain. The secondary outcome induces PCA usage on POD 1 and morphine equivalent, which is calculated based on the narcotics administered in the PACU and post-operative day 1 (POD 1) with established conversion [14]. Additionally, the incidence of post-operative nausea and vomiting (PONV) was documented, and the time to first pain medication administration was measured from the end of surgery until the patient's initial analgesic intake.

Sample size determination

A post-hoc power analysis remains informative to provide estimations of the minimal detectable effect size based on our available study sample [15]. Utilizing a two-independent sample t-test and assuming the statistical power = 0.9 and a two-tailed alpha = 0.05, our available analytical sample N = 68 (34 each group) could detect a minimal effect size of mean difference (MD = 1.4) in pain scores, assuming its standard deviation (SD) = 1.8. This effect size was considered moderate, and it was clinically meaningful. Our study sample size was also in accordance with a previous trial composed of a similar population and intervention [16].

Statistical analysis

Descriptive statistics were reported using MD and SD or median and 25th/75th percentiles (i.e., Q1/Q3) for continuous variables and frequencies and percentages for categorical variables. Baseline demographics, procedural level variables, and outcomes were summarized and compared using standardized mean differences (SMD). We used non-parametric testing such as Fisher's exact test and Wilcoxon test to make between-group comparisons. For assessing the differences in study outcomes between patients in the RA group and those in the NB group, we performed both crude and adjusted multivariable generalized linear regression analyses with proper link functions (e.g., normal, logit). Data transformation in the outcomes (e.g., log transformation) was performed to account for the non-normal distribution of study outcomes such as VAS scores. The list-wise deletion method was primarily employed to handle missing data in primary and secondary analyses. In sensitivity analysis, we also conducted the multivariate imputation by chained equations (MICE) and performed the regression analyses on the primary outcome. All statistical tests are two-tailed, and the alpha was set to 0.05. Statistical analyses were performed using RStudio and R statistical software (RStudio PBC, Boston, MA).

## Results

**Figure 1:**
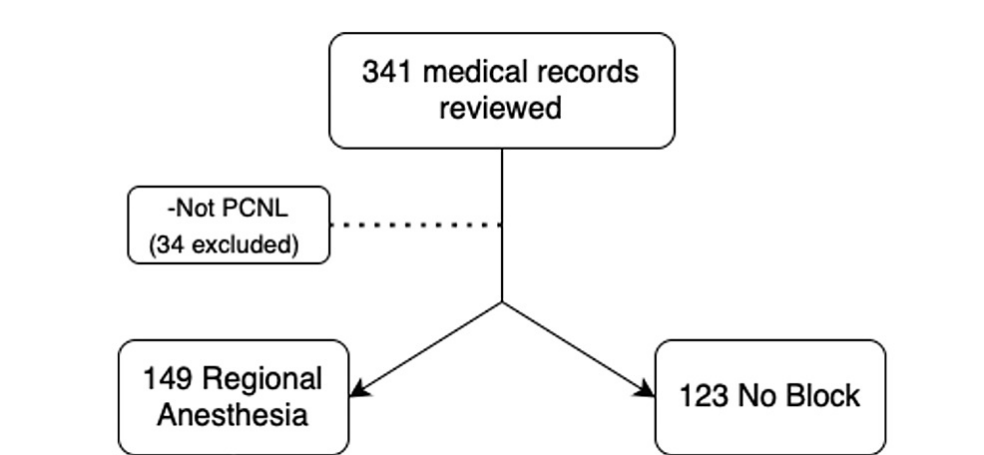
Subjects' chart review flowsheet Image from the author. The number of cases (N) is displayed as total counts.

This retrospective study, conducted from July 2014 to April 2016, initially included 341 subjects. After excluding non-PCNL patients and accounting for missing data, we ultimately analyzed 275 subjects. Among these, 149 subjects received preoperative single-shot regional anesthesia aimed at postoperative pain control, while 123 received none. Patients we excluded from the analysis primarily consisted of non-PCNL patients and those with missing data, consisting of 19% of our initial sample (Figure 1).

There are no significant demographic differences between the groups, except that the NB group exhibited a higher ASA status, and a history of chronic pain was more common in the RA group (Table 1).

**Table 1:** Demographic characteristics of included patients The table presents summary statistics for our sample, separately for patients not receiving regional anesthesia (NB) or patients who did receive regional anesthesia (RG). P refers to the statistical significance of differences between the two groups. A p-value < 0.05 is considered statistically significant. Values are given as N (%) or means±SD. NB: No-Block; RA: Regional Anesthesia; BMI: Body Mass Index; ASA: American Society of Anesthesiologists

Characteristic	N	NB (N=123)	RG (N=149)	p-value
Age (years)	272	56.41±13.18	58.36±14.42	0.250
Male sex (%)	272	75 (61%)	74 (49.7%)	0.081
Weight (kg)	265	82.26±25.65	85.17±21.79	0.319
Height (in)	252	66.40±4.28	66.58±4.08	0.742
BMI	252	28.74±8.52	29.83±6.93	0.264
ASA Score (%)	269	-	-	0.011
1	11	4 (3.3%)	7 (4.8%)	n/a
2	154	58 (47.5%)	96 (65.3%)	n/a
3	103	59 (48.4%)	44 (29.9%)	n/a
4	1	1 (0.8%)	0	n/a
Chronic Pain History (%)	272	1 (0.8%)	10 (6.7%)	0.032

Primary outcome analysis (Tables 2-3) indicated no significant difference in pain scores within the PACU, as assessed by the VAS. Both the minimum (N = 201, SMD = 0.207; adjusted regression analysis: log-transformed beta = 0.96, 95% CI = -0.02 to 1.9; p = 0.054) and maximum (N = 201, SMD = 0.244, adjusted regression analysis: log-transformed beta = 0.15, 95% CI to -1.0 to 1.4; p = 0.800) pain scores did not differ significantly between groups, after adjusting for age, sex, BMI, and American Society of Anesthesiologist (ASA) status. In the additional MICE sensitivity analysis, both minimal and maximum PACU pain scores exhibited significant differences (p < 0.001 and p = 0.037, respectively). We considered this significant difference might not be reliable and was likely due to the high missing rate between groups (i.e., over 40% missingness in the RA group and approximately 25% in the NB group), and our complete-case primary analysis results shall also be interpreted with caution considering the high missing rate in pain outcomes.

**Table 2:** Outcome parameters comparing both groups The table presents the primary and secondary outcomes for our sample (N), separately for patients not receiving regional anesthesia (NB) or patients who did receive regional anesthesia (RG). P refers to the statistical significance of differences between the two groups. A p-value < 0.05 is considered statistically significant. 95% CI are shown alongside mean±SD. Values are given as n (%) or means±SD. NB: No-Block; RA: Regional Anesthesia; PCA: Patient-Controlled Analgesia; PACU: Post-Anesthesia Care Unit; LOS: Length of Stay; POD: Post-operative day

Characteristic	N	NB group	RA group	p-value	95% CI
PCA usage (%)	272	14 (11.2%)	5 (3.4%)	0.019	0.07, 0.55
Maximum PACU Pain VAS	201	3.47±3.44	4.30±3.36	0.102	-0.54, 0.05
Minimum PACU Pain VAS	201	0.42±1.39	0.74±1.71	0.178	-0.50, 0.08
PACU Total Morphine Used (mg)	155	8.4±8.1	9.4±7.4	0.447	-0.46, 0.20
POD 1 Total Morphine Used (mg)	230	7.10±14.46	6.98±9.08	0.938	-0.25, 0.27

**Table 3:** Primary outcomes adjusted analysis This table represents the regression analysis for the primary outcomes with the crude model and the adjusted model for each variable (age, sex, BMI, or ASA). P refers to the statistical significance of differences between the two groups. A p-value < 0.05 is considered statistically significant. Values are given as 95% confidence interval PACU: Post-Anesthesia Care Unit; VAS: Visual Analogue Scale; BMI: Body Mass Index; ASA: American Society of Anesthesiologists

Variable	Maximum PACU Pain VAS				Minimum PACU Pain VAS			
	Crude Model		Adjusted Model		Crude Model		Adjusted Model	
	95% CI	p-value	95% CI	p-value	95% CI	p-value	95% CI	p-value
Regional Anesthesia (Yes)	-0.22, 2.1	0.110	-1.0, 1.4	0.800	0.02, 1.8	0.046	-0.02, 1.9	0.054
Age	-	-	-0.04, 0.05	0.828	-	-	-0.06, 0.01	0.216
Sex (Female)	-	-	-0.37, 1.8	0.193	-	-	-0.47, 1.3	0.350
BMI	-	-	--0.05, 0.10	0.493	-	-	-0.06, 0.06	0.981
ASA	-	-	-3.5, -1.1	< 0.001	-	-	-0.92, 1.1	0.883

Secondary outcomes (Table 4) revealed a significant difference in the PCA use on the first postoperative day (adjusted logistic regression analysis: OR = 0.19, 95% CI = 0.05-0.60; p = 0.008), favoring the NB group. However, the morphine usage in both PACU (p = 0.330) and first POD (p = 0.613) was not statistically different between the two study groups based on our multivariable linear regression analyses. All adjusted regression analysis results were mostly consistent with the crude analyses, except for minimal VAS scores (i.e., the crude analysis was significant but not in the adjusted analysis), and detailed results, including parameter estimates and corresponding 95% CIs, were reported in Tables 2-4.

**Table 4:** Secondary outcomes adjusted analysis This table represents the regression analysis for the secondary outcomes with the crude model and the adjusted model for each variable (age, sex, BMI, or ASA). P refers to the statistical significance of differences between the two groups. A p-value < 0.05 is considered statistically significant. Values are given as 95% confidence interval. PACU: Post-Anesthesia Care Unit; VAS: Visual Analogue Scale; BMI: Body Mass Index; ASA: American Society of Anesthesiologists

Variable	PCA Usage				Morphine Consumption PACU				Morphine Consumption POD 1			
	Crude Model		Adjusted Model		Crude Model		Adjusted Model		Crude Model		Adjusted Model	
	95% CI	p-value	95% CI	p-value	95% CI	p-value	95% CI	p-value	95% CI	p-value	95% CI	p-value
Regional Anesthesia (Yes)	0.09, 0.73	0.015	0.05, 0.60	0.008	-1.6, 3.5	0.447	-1.4, 4.0	0.330	-3.2, 3.0	0.938	-4.2, 2.5	0.613
Age	-	-	0.95, 10.3	0.499	-	-	-0.21, -0.02	0.016	-	-	-0.22, 0.02	0.098
Sex (Female)	-	-	0.29, 2.56	0.816	-	-	-3.5, 1.4	0.396	-	-	-3.1, 3.6	0.879
BMI	-	-	0.98, 1.11	0.163	-	-	-0.15, 0.17	0.881	-	-	-0.04, 0.39	0.105
ASA	-	-	0.10, 1.33	0.159	-	-	-1.0, 4.7	0.203	-	-	-5.2, 2.0	0.371

## Discussion

Controlling post-operative pain after PCNL is an area of interest due to the morbidity it causes and the burden it places on healthcare [4,17,18]. Early studies and reports have consistently reported the effectiveness of epidural catheters on post-PCNL pain control and reducing overall post-operative opioid use [9,19] without negatively affecting surgical outcomes compared to the traditional general anesthetic [20,21]. However, epidural analgesia usage is limited in PCNL, as thoracic epidural might increase the length of hospital stay and ambulation [22].

Peritubal infiltration of local anesthetics has also been used as another method for post-PCNL pain control but has less analgesic properties in comparison to TPB [23,24], the latter being an effective alternative to the epidural catheter in providing unilateral coverage for post-PCNL pain, as evidenced by a reduction in post-operative opioid consumption [16,25,26] and comparatively less effect on hemodynamic parameters, less risk to the neuraxis structure, and early ambulation.

The effectiveness of using PVB on post-PCNL pain control has been observed by Borle et al., who placed a unilateral PVB catheter at the level of T9-10 in 50 patients and observed a significant decrease in the amount of intraoperative fentanyl use and VAS score [27]. A recent randomized controlled trial by Yaman et al. [28] demonstrated similar pain-control results up to four hours after surgery, favoring the PVB group. Additionally, their control group used more opioids for pain relief and presented lower satisfaction scores. The finding was also supported by another RCT by Wagaskar et al. who found lower VAS scores at six hours post-procedure and lower requirement of rescue analgesia in the intervention group. Finally, a recent meta-analysis by Tan et al. [29] reported that the PVB reduced the analgesic consumption of additional analgesics and prolonged the time to the first analgesic requirement compared to the control. Although the cited evidence has been in favor of using PVB for post-PCNL pain control, predefined and controlled samples may not accurately represent the diverse population of patients that treatment is intended for. This lack of representation can limit the generalizability of the trial's findings to broader populations. In addition, RCTs that yield positive results are more likely to be published, while those with negative or inconclusive outcomes may remain unpublished. This publication bias can skew the overall perception of treatment effectiveness [30]. 

In our study, the use of the regional technique was not associated with significantly lower PACU pain scores, changes in PONV, or morphine consumption in the PACU. Although we had 30% more usage of PCA in the NB group, the total narcotic usage was not different between the two groups.

**Figure 2:**
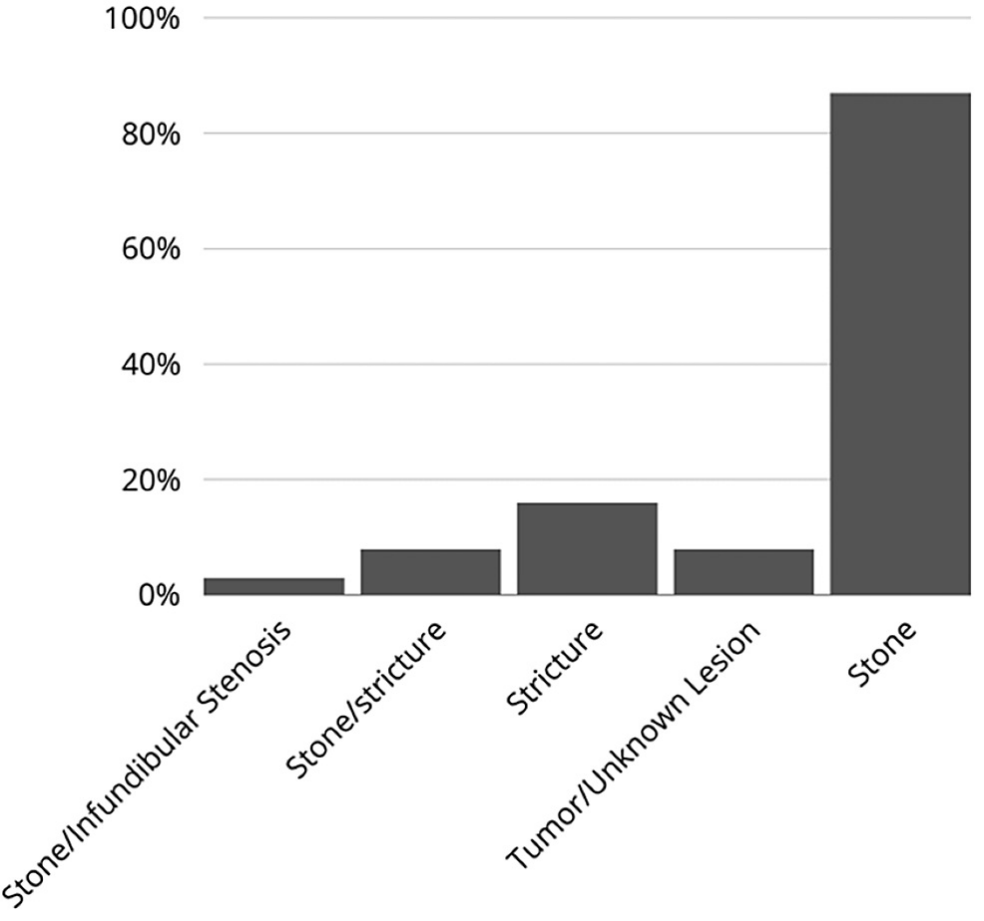
Surgical indications Image from the author. The data are presented as percentages (%) of each subgroup of procedures that constitute the total sample.

**Figure 3:**
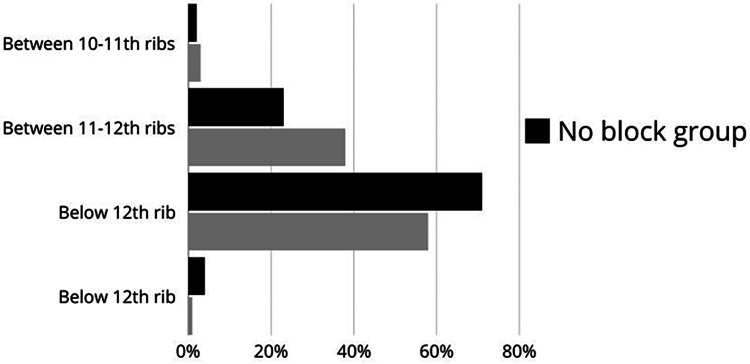
Surgical incision site Image from the author. The data are presented as percentages (%) of the final height at which the block was performed.

We believe this lack of efficacy of PVB in providing post-PCNL analgesia is related mostly to surgical confounding factors. The relative distribution of the indication of surgery and place of trocar is reflected in Figures 2-3, respectively. While PCNL is typically used for nephrolithiasis, it has been used for relieving strictures and or even tumor resection [31]. The complexity of these surgical procedures requires the trocar insertion site to be as low as below T12 (Figure 3). While most of the PVB was done at the T7-T9, this injection may not cover enough to provide analgesia for our patient population.

There are several limitations to this study regarding VAS and pain scores: Pre-existing pain is infrequently recorded in perioperative assessments, and the VAS may be influenced by factors other than acute pain intensity [32]. Pain at rest but not with movement was not recorded. The location of pain was also inconsistently documented, so pain outside of the PVB coverage was not detailed. Inappropriate administration of pain medication (i.e., used to treat agitation and/or shivering). Discharge from the PACU was affected by other factors besides patients’ readiness (such as availability of the floor bed). Finally, retrospective studies are susceptible to data bias, and the information gathered during and after the surgery was not entirely complete.

This is a retrospective study and, while valuable for exploring associations and trends, has inherent limitations that must be considered. Firstly, it is reliant on pre-existing data, often collected for purposes other than the specific research question at hand, leading to potential inaccuracies or missing information. Additionally, retrospective studies are susceptible to selection bias, as researchers have no control over the selection criteria or data collection methods. There may also be confounding variables that are not adequately accounted for, undermining the ability to establish causal relationships between variables. Furthermore, factors such as our study's single-center design, the homogeneity of our patient population, and the specific PVB administration techniques directly influence the generalizability of our results. To validate these results' external validity, future research must replicate them in varied clinical settings and with diverse populations. Despite these limitations, retrospective studies can provide valuable insights, particularly when used in conjunction with other study designs.

## Conclusions

The retrospective analysis did not reveal any discernible advantage in pain management associated with the use of PVB for PCNL analgesia. The intricacy of the surgery and the chosen approach may serve as significant confounding variables affecting the efficacy of PVB for this specific procedure. Future investigations with larger sample sizes and meticulous control for surgical indications and complexity are imperative to accurately assess this block's efficacy in post-PCNL surgery.
